# An Introduction to Hospital Practice, in Various Complaints; Being a Clinical Report of Fever, Gout, Rheumatism, Cholera, Jaundice, Erysipelas, Insanity, &c., and Diseases of the Chest and Heart; with Remarks on Their Pathology and Treatment

**Published:** 1836-04

**Authors:** 


Art. II.—*An Introduction to Hospital Practice, in various Complaints; being a Clinical Report of Fever, Gout, Rheumatism, Cholera, Jaundice, Erysipelas, Insanity, &c., and Diseases of the Chest and Heart; with Remarks on their Pathology and Treatment.* By C. J. B. Aldis, m.a., m.b., and l.m., Inceptor Candidate of the Royal College of Physicians.—*London*, 1835. 8vo. pp. 125.

This is a collection of cases reported, with few exceptions, by Dr. Aldis, whilst a student at St. George's Hospital, in 1832-33. Remarks on morbid anatomy and treatment are appended to the cases, and they are introduced by a preface setting forth the merit and aims of clinical medicine. The plan of such a work is similar to that of the Clinique Médicale of M. Andral, who, as a young man, reported the cases of M. Lerminier. But the mode in which it is executed is widely different. It would have been a subject of congratulation to see the resources of a large hospital turned to account by an advanced student, who would have taken upon himself the laborious task of carefully reporting a large body of cases, and of minutely analysing them. By such a plan a young man might add to or increase the exactness of our knowledge of disease, making use of the practical experience of his elders, which might otherwise have been lost. But an undertaking of this character, to be useful at all, must be entered into with the diligent spirit of an Andral or Louis. The size of Dr. Aldis's book, and the numerous diseases displayed on the title-page, shew at once that this is not the plan which he has chosen. In a thin octavo of 125 pages, he has attempted to illustrate, by cases, the principal diseases which come under the care of the physician.

In the preface, Dr. Aldis tells us that "Clinical Medicine is progressing but slowly in this country, and numerous valuable facts which are constantly occurring, sink into oblivion," (p. ix.); and that "the management of diseases has been much neglected by writers on Clinical Medicine," &c. (p. xvi.) These charges are unfair, because unmerited. Every one conversant with our present standard medical works and transactions, is aware that clinical medicine is making rapid progress; that it never was cultivated so generally; and that the distinguishing characteristic of British medical writers, is the full and careful way in which they discuss the management of diseases. That many valuable facts sink into oblivion, is true; but we will give our readers an opportunity of judging whether Dr. Aldis has learned the secret of preserving them from obscurity. We have alluded to Dr. Aldis's censures on others, from a feeling that the pretensions of an author should be duly estimated in forming any judgment on his works. We shall select the cases on diseases of the lungs, as Dr. Aldis, in his preface, fully recognizes the utility of the stethoscope, which he states has introduced an accuracy in diagnosis that Cullen thought would never be possible. The first case (case 76) is called "Acute Bronchitis."

"Mary Ann D., æt. 28, servant, admitted April 27, 1832, complaining of very severe pain of the left side of the chest, shooting across to the other side, with very frequent cough, accompanied by expectoration, which is of a dark brick colour. Was seized five days previously with pain under the left breast, and cough, for which she was ordered to apply a blister twice.

"℞ Haust. Salin., Haust. Nitri, āā ʒvj.; Pulv. Ipec. c. gr. iij. 6tis horis.

"℞ Magn. Sulph., ʒvj.; Infus. Rosæ, ℥iss. statim. (D. parcissima.)

"28. Hirud. viij. lateri et postea Empl. Canth.
"℞ Pulv. Ipec. c. gr. v. h. n.
"Haust. Salin. c. Oxym. Scillæ ʒj., Magn. Sulph. ʒss. t. d.
"29. P.
"May 2. Pulse 100; tongue clean and moist. Fish. P.
"6. Convalescent. (D. ordinaria.) Haust. Sennæ crâs.
"8. Cured." (Page 101.)

The mode of attack, the probable cause, the state of the circulation and of the tongue, are entirely omitted; and the chest symptoms are not alluded to after the first day. No evidence is even given that bronchitis existed: the dark brick-coloured expectoration and pain would rather lead to the supposition of pneumonia.

The second case is one of chronic bronchitis; despatched in the same space, and equally unimportant. The third is of the same disease, in which no other pulmonary symptoms are alluded to than pain and expectoration. The fourth is called "Chronic Bronchitis, with diseased heart;" where the usual symptoms of chronic bronchitis are mentioned, but not a single symptom peculiar to disease of the heart. Asthma is illustrated by one case, occupying a dozen lines; and in the "Remarks" we learn that "Asthma merely means difficulty of breathing," (p. 105): as if asthma and dyspnœa were synonymous terms. One case also is reported under the head pulmonary consumption, to prove that the patient was once bled with relief. Two cases are reported of pneumonia: in the first, instead of the previous symptoms, it is carelessly stated that there was inflammation of the *chest* of four days' duration; and the only pectoral symptoms mentioned on admission are—"inspires with difficulty, but without pain:" no notice being taken of the expectoration until three days afterwards, when it is announced to be "loose."

It is unnecessary to continue an analysis of cases which will only afford further illustrations of careless and superficial reporting. In only six cases out of fifteen was the stethoscope employed; and the information reported is too vague to be useful, and often so inconclusive as to afford no evidence of the benefit of the instrument in the detection of disease. Thus, we are told there was on the same spot "a very dull sound, and crepitating râle," (p. 110); and in another case (p. 113) where, after death, the ventricles were found very much dilated, and the mitral valve ossified, no information was gained by auscultation. Percussion alone seems to have been employed in a case reported as pleurisy; and in a case of phthisis, the stethoscope is stated to have been placed beneath the acromial extremities of the clavicles.

In the account given of some of the cases of fever, the daily report is sometimes limited to a list of the medicines ordered, the symptoms being wholly left out. Dr. Aldis divides fever into three periods: the premonitory, the congestive, and the period of lesions. Of the congestive period he says, "Active treatment is always requisite during the second stage. If there be *determination of blood* to the *head or chest*, we must employ *general or local* bloodletting." A few lines further on, we are told "If there be *determination* to the *chest or abdomen*, general or local bloodletting may be necessary." The period of "lesions" is treated with equal dogmatism; as if, indeed, the author's object had been to illustrate the clinical imperfections denounced with so calm an air of superiority in his preface.

In a note at page 7, the author makes a remark on the importance of attending to the condition of the bladder in fever; and mentions a case of a lady to whom he was "called," in whom it had been overlooked: but Dr. Aldis mentions this as if it were his original suggestion.

The remarks appended to each set of cases, and which "are confined to the pathology and management of the diseases in question," are in character with the cases: superficial, and even puerile. No one will condemn this criticism as harsh, after reading the following specimen, which we copy because it is short, and completely illustrates Dr. Aldis's style and matter.

"*Infantile Remittent Fever.*—Remarks. Infantile fever appears always to arise from a disordered state of the bowels. Sometimes the skin is extremely hot. The child may pick at the bed-clothes, but not as adults are accustomed to do, in the latter periods of fever. Children take hold of something prominent, like a knot in the counterpane. Saline draughts are of little use. Purgative medicines are most to be depended upon. It is necessary to be very careful about diet; respecting which, practitioners are very frequently foiled in private practice. The disease may terminate in marasmus, in which the mesenteric glands become enlarged, and the abdomen tumid. The child represents a little old person." (P. 34.)

It would not be easy to comprehend, in the same space, less matter of any value, or to express so many trivial details more feebly. The following is called the "Pathology" of pulmonary consumption:

"The lungs do not collapse. There is more than the usual quantity of black pulmonary or carbonaceous matter. Vomicæ, lined with a membrane, are generally found at the apices of the lungs. Small granular bodies, termed miliary tubercles, are observed. The pleura may be perforated, and occasion pneumato-thorax. A portion of the lung may be condensed with tubercular infiltration. A cicatrix is sometimes met from the cohesion of the sides of the vomicæ. Earthy concretions, composed of the phosphate or carbonate of lime, (the former more frequently than the latter,) are occasionally discovered. Portions of the intestine may be ulcerated or perforated." (P. 106.)

This is not selected (as might be supposed,) because particularly faulty: it is a faithful sample of the whole. If we were to judge by this quotation of Dr. Aldis, we might suppose him unacquainted with the progress of tubercles, of the situation they successively occupy in the lungs, and of the tubercular nature of vomicæ; in short, he might be thought utterly unacquainted with the researches of Bayle, Laennec, and Louis, on this subject. Indeed, we feel at a loss to conjecture from what sources Dr. Aldis derived his medical knowledge, and particularly so when he discusses, as he often does in seeming earnest, opinions which he attributes to "pathologists and physicians."

There are numerous indications of a want of perception of the actual state of medicine, or of the kind of information which an Introduction to Hospital Practice should contain. Thus we are told, with the amusing air of gravity with which the author seems to consider himself as enunciating novelties, that "Quinine is considered an excellent antiperiodic medicine." (P. 37.) "Incisions" (as recommended by Mr. Hutchinson and Mr. Lawrence in erysipelas,) "are not employed on the face." (P. 34.) "Much might be said on the moral treatment of insanity; and whenever I have visited establishments where this was adopted, with medical advice, I have considered that every thing was done to secure the recovery of the curable, and to promote the comfort of the incurable." (P. 84.) "In the diagnosis of serous effusions into the chest, I have seen percussion alone fallacious." (P. 118.) Nothing surely can be less suited to the limited knowledge here betrayed than the tone adopted by the author.

The pathology of insanity is summarily disposed of. "It has generally been supposed that some peculiarity of the brain disposes to madness; but this peculiarity has never been clearly ascertained. *Morbid appearances are seldom discovered in the brain.*" A few more remarks are added, indicative, we regret to observe, of the fullest ignorance of the researches of Bayle, Calmeil, Foville, and Esquirol, in France, and of all the most accredited writers on insanity in our own country.

There is as little to commend in the composition and style of Dr. Aldis's Introduction to Hospital Practice, as in the matter. The sentences in general have so little connexion with each other, that they might constantly be transposed without altering the sense. They are usually bald and inelegant, frequently ambiguous, and occasionally ungrammatical. The following are either ungrammatical or unintelligible:

> "Such writers inculcate the necessity of observation, and defend us from obscure hypothesis. In them, observation and reason are united; the one is not alloyed by preconceived opinions, the other is modified by an attention to facts." (P. xi.)

And this passage, it is to be observed, is republished from the Medical Gazette; we presume with the deliberate approval of the author. Again:

> "Hypochondriasis, and the early symptoms of insanity, are unfortunately often ridiculed in families, not from deficiency of feeling on their part, but with a view to dissipate ennui, or any erroneous impression which might absorb the mind of the person affected; at the same time being unacquainted with the nature of the complaints."

It is an unpleasant part of our duty to notice a publication of this character. The very object of it is somewhat ænigmatical. Young physicians are, we know, often exhorted by dull advisers to "write a book;" and it would seem as if, following such injudicious exhortation, Dr. Aldis had emptied his hospital case-book. To entitle such a work, the imperfect note-book of a student, an Introduction to *Hospital* Practice, was certainly to give it a name to which it has no pretensions.

If Dr. Aldis's ambition had been better directed, a few more years of better observation in the noble institution which furnished him his cases, *might* have enabled him to publish a monograph, at least, which would have been accepted with gratitude by all medical readers. As it is, we can but trust that this Introduction to Hospital Practice will not reach the opposite side of the Channel. We can conceive no mortification greater than the exhibition of such a production in a French hospital as a specimen of English *clinique*. Nor is it fortunate, at the present juncture, that such a work is published by a physician to whose name the appended letters "M.A., M.B., and L.M.," and the title of "Inceptor Candidate of the Royal College of Physicians, London," would have made us hope for better things.

